# Distinct neuroanatomical correlates of DBQ-derived illegal, irritable, and aggressive driving tendencies in healthy adults: an exploratory structural MRI study

**DOI:** 10.3389/fnhum.2026.1907749

**Published:** 2026-08-28

**Authors:** Kaechang Park, Handityo Aulia Putra

**Affiliations:** 1 Research Organization for Regional Alliance, Kochi University of Technology, Kami, Japan; 2 Department of Decoded Neurofeedback, Computational Neuroscience Laboratory Group, Advanced Telecommunications Research (ATR) Institute International, Kyoto, Japan; 3 Faculty of Health Sciences, University of Kochi Health Sciences, Kochi, Japan; 4 Department of Electrical, Electronics, and Information Engineering, Nagaoka University of Technology, Nagaoka/Niigata, Japan; 5 Department of Cyber Defense Engineering, Republic of Indonesia Defense University, Bogor, West Java, Indonesia

**Keywords:** brain checkup, driving behavior questionnaire, hazardous driving, structural MRI, voxel-based morphometry

## Abstract

**Background:**

Hazardous driving encompasses multiple forms of aberrant behavior that have been associated with increased crash involvement and unsafe driving outcomes. Although, structural MRI studies have linked regional brain morphology to driving performance and driving-related errors, evidence regarding the neuroanatomical correlates of distinct Driving Behavior Questionnaire (DBQ)-derived hazardous driving tendencies remains limited in large community-based samples of healthy adults.

**Methods:**

We analyzed data from 1,070 individuals undergoing routine brain health checkups who completed the DBQ and structural MRI. Three theoretically derived hazardous driving domains were examined: illegal, irritable, and aggressive driving. Participants were classified into higher-tendency and reference groups using predefined domain-specific thresholds. Multivariable logistic regression models were constructed using age, sex, driving frequency, and z-transformed regional brain volumes as explanatory variables. Stepwise variable selection based on the Akaike Information Criterion (AIC) was applied. Leave-one-out cross-validation (LOOCV) was used to evaluate model robustness and the stability of selected neuroanatomical correlates.

**Results:**

Male sex was consistently associated with all three hazardous driving domains, whereas younger age was specifically associated with aggressive driving. The multivariable models demonstrated moderate discriminative performance, with mean AUCs under LOOCV of 0.735 for illegal driving, 0.757 for irritable driving, and 0.796 for aggressive driving. Illegal driving was primarily associated with insular and sensorimotor regions, including the precentral and postcentral gyri. Irritable driving was associated with a broader network involving thalamic, striatal, temporolimbic, and posterior cortical regions. Aggressive driving showed a more focused pattern centered on the caudate, putamen, and right lingual gyrus. Notably, the right caudate and right lingual gyrus were common to both irritable and aggressive driving models. The direction of these associations remained stable across LOOCV iterations.

**Conclusions:**

Distinct DBQ-derived hazardous driving tendencies were associated with partially divergent yet overlapping regional brain volumetric patterns. These findings suggest that illegal, irritable, and aggressive driving may reflect partially distinct neurobehavioral processes related to risk regulation, emotional reactivity, and habitual aggressive responding. Although independent replication is required, these hypothesis-generating findings provide an initial neuroanatomical framework linking brain structure to distinct hazardous driving tendencies in neurologically healthy adults.

## 1 Introduction

Traffic crashes remain a major public health and transportation safety challenge worldwide. Although environmental, roadway, and vehicle-related factors contribute substantially to crash occurrence, stable individual differences in driving behavior also play an important role in determining crash risk. Self-reported aberrant driving tendencies have therefore become an important focus of traffic safety research (Reason et al., 1990; de Winter and Dodou, 2010; Cordazzo et al., 2014).

The Driving Behavior Questionnaire (DBQ) is one of the most widely used instruments for assessing habitual aberrant driving behaviors. Since its original description, the DBQ has been adapted and validated in multiple settings and populations, and its dimensions have shown modest but consistent associations with self-reported crash involvement (Reason et al., 1990; de Winter and Dodou, 2010; Cordazzo et al., 2014; Parker et al., 1995). At the same time, hazardous driving is not a unitary construct. Different forms of aberrant driving may arise from distinct psychological motivations and behavioral tendencies. In the present study, we focused on three theoretically derived hazardous driving domains: illegal driving (deliberate rule violation), irritable driving (frustration-related driving), and aggressive driving (hostile or competitive driving). These domains are likely to reflect partially different psychological processes and may therefore have partially different neurobiological substrates.

Previous efforts to evaluate hazardous driving have often relied on behavioral testing, driving simulation, or self-report instruments. Several structural MRI studies have linked regional gray matter volume to driving-related outcomes. Smaller premotor gray matter volume has been associated with poorer executive function and increased driving errors (Sakai et al., 2012), whereas machine-learning analyses have identified cortical gray matter patterns distinguishing unsafe from safe older drivers (Yamamoto et al., 2020). Regional brain volumes have also been associated with driving safety behaviors (Park et al., 2024), driving performance (Putra et al., 2025), and ADHD-related traits linked to real-world crash involvement (Putra et al., 2023). Collectively, these findings suggest that individual differences in brain structure may contribute to hazardous driving through both cognitive performance and stable behavioral traits.

These prior studies focused mainly on older drivers, executive function, objective driving safety performance, or crash-related outcomes rather than DBQ-derived subtypes of illegal, irritable, and aggressive driving in a large cohort of neurologically healthy adults. Voxel-based morphometry (VBM) and related regional volumetric methods provide a practical framework for addressing this gap in large community-based cohorts in which structural MRI data and behavioral assessments are available (Ashburner and Friston, 2000; Scarpazza and De Simone, 2016).

To our knowledge, no large-scale structural MRI study has systematically examined whether distinct DBQ-derived hazardous driving subtypes are associated with different neuroanatomical patterns in neurologically healthy adults. Accordingly, the present study aimed to determine whether these three DBQ-derived hazardous driving domains exhibit distinct regional gray matter volume profiles in a large cohort of neurologically healthy adults.

From a neurobehavioral perspective, regions implicated in salience detection, cognitive control, frontostriatal regulation, and visuospatial processing are plausible candidates for involvement in hazardous driving. In particular, the insula has been linked to salience processing and behavioral regulation, whereas frontostriatal regions including the caudate have been associated with impulsivity and response control (Menon and Uddin, 2010; Dang et al., 2016; Elliott et al., 2023). These considerations support the hypothesis that different hazardous driving subtypes may be associated with partially distinct but overlapping neuroanatomical systems. Specifically, we anticipated that illegal driving would be more closely associated with neural systems involved in risk monitoring and behavioral restraint, whereas irritable and aggressive driving would show stronger associations with regions implicated in emotional regulation and frontostriatal behavioral control. These hypotheses were intended to provide a conceptual framework for interpretation rather than to test highly specific regional predictions.

## 2 Methods

### 2.1 Participants

**Figure 1:**
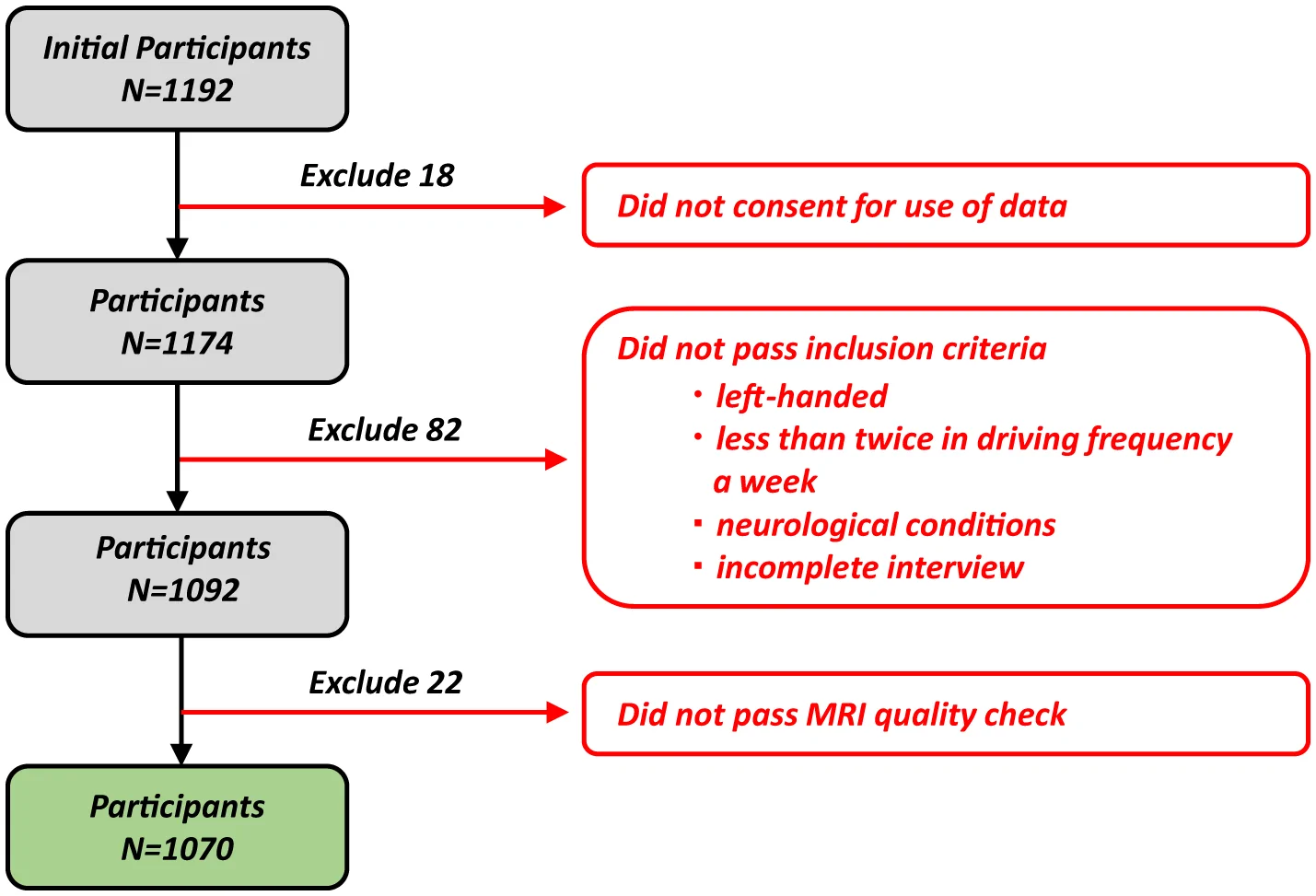
Flowchart of participant selection. The study cohort was derived from individuals who underwent brain checkups in 2013 and completed the Driving Behavior Questionnaire (DBQ). Participants who did not consent to research use of their data, did not pass MRI quality control, or did not meet the inclusion criteria were excluded. Additional exclusions were applied to participants with incomplete DBQ responses and to those with unsuccessful volumetric segmentation. The final analytic sample comprised 1,070 neurologically healthy adults.

The study cohort comprised individuals who underwent brain checkups in 2013, including the DBQ and structural MRI. Participant selection is summarized in Figure 1. We excluded individuals who: (1) did not consent to the research use of their data; (2) did not meet the predefined inclusion criteria (e.g., left-handedness, low driving frequency, or neurological conditions); (3) failed MRI quality control or subsequent volumetric segmentation. Additionally, participants with incomplete DBQ responses or unsuccessful volumetric analysis were excluded. The final analytic sample consisted of 1,070 neurologically healthy adults (mean age 53.08 ± 8.83 years; 46.9% male). The participant received a comprehensive explanation of the consent form from K.P., delivered both verbally and in written documentation.

### 2.2 DBQ-derived hazardous driving domains

Three behavioral domains were defined *a priori*: illegal, irritable, and aggressive driving. The illegal domain included items 7, 34, 35, 43, and 48; the irritable domain included items 16, 18, 25, 26, 28, and 29; and the aggressive domain included items 14, 36, 40, and 45. These domains were defined a priori to represent three conceptually distinct forms of hazardous driving behavior: deliberate rule violation (illegal driving), frustration-related driving (irritable driving), and hostile or competitive driving (aggressive driving).

The original Driving Behavior Questionnaire (DBQ) contains multiple categories of aberrant driving behaviors, including violations, errors, and lapses. The purpose of the present study was not to re-examine the psychometric factor structure of the DBQ, but rather to investigate whether theoretically distinct forms of hazardous driving behavior are associated with different neuroanatomical correlates. Accordingly, three hazardous driving domains were defined a priori based on the behavioral characteristics represented by individual DBQ items. Item selection was guided by conceptual considerations derived from traffic psychology and behavioral theory rather than data-driven factor analysis (Table 1).

**Table 1:** Conceptual definitions of the three hazardous driving domains.

Domain	Conceptual definition	DBQ items
Illegal	Deliberate violation of formal traffic rules despite awareness of legal and safety consequences	7, 34, 35, 43, 48
Irritable	Hazardous driving behaviors primarily driven by impatience, frustration, or emotional tension in traffic situations	16,18, 25, 26, 28, 29
Aggressive	Interpersonally competitive, hostile, or confrontational driving behaviors directed toward other road users	14, 36, 40, 45

The Illegal domain included behaviors representing deliberate violations of traffic laws and regulations despite awareness of potential legal or safety consequences. Examples included intentional speeding, red-light violations, driving against traffic on a one-way street, driving under the influence of alcohol, and failure to obey a stop sign.

The Irritable domain comprised behaviors reflecting impatience, frustration, and emotionally driven risk-taking in response to traffic situations. These behaviors were characterized by tension arising from perceived delays, slow-moving traffic, or situational frustration, leading to potentially hazardous driving decisions. Items such as failure to yield (Item 16), shoulder overtaking (Item 18), and crossing at a changing traffic signal (Item 29) were included in the irritable domain because these behaviors were interpreted as reflecting impatience and frustration arising from traffic-related delays rather than primarily hostile intent or deliberate rule violation. The classification was based on the predominant psychological motivation underlying each behavior rather than on its legal status alone.

The Aggressive domain consisted of behaviors involving interpersonal competition, hostility, or confrontational interactions with other road users. These items reflected intentional actions directed toward other drivers and included competitive driving, hostile attitudes toward specific driver groups, and behaviors suggesting dominance-seeking or antagonistic intent.

These domains were conceptualized as theoretically derived behavioral phenotypes rather than psychometric factors. The objective was to examine whether neuroanatomical variation was differentially associated with distinct forms of hazardous driving behavior that may arise from different underlying psychological processes, including rule violation, emotional reactivity, and interpersonal aggression.

Rather than attempting to reproduce previously reported DBQ factor structures, the present classification was intentionally designed to represent conceptually distinct hazardous driving phenotypes that could be examined from a neuroanatomical perspective. We considered that illegal, irritable, and aggressive driving reflect different behavioral processes involving rule violation, frustration-related emotional responses, and hostile interpersonal behavior, respectively. This theory-driven approach was adopted to facilitate biological interpretation of distinct patterns of regional brain morphology.

The remaining DBQ items were not included because they represented behaviors outside the conceptual scope of the present three-domain classification or did not predominantly reflect illegal, irritable, or aggressive driving tendencies.

The present classification was intended to provide biologically interpretable behavioral phenotypes for neuroimaging analysis rather than to redefine the psychometric structure of the DBQ.

### 2.3 Two-step dichotomization of domain scores

**Figure 2:**
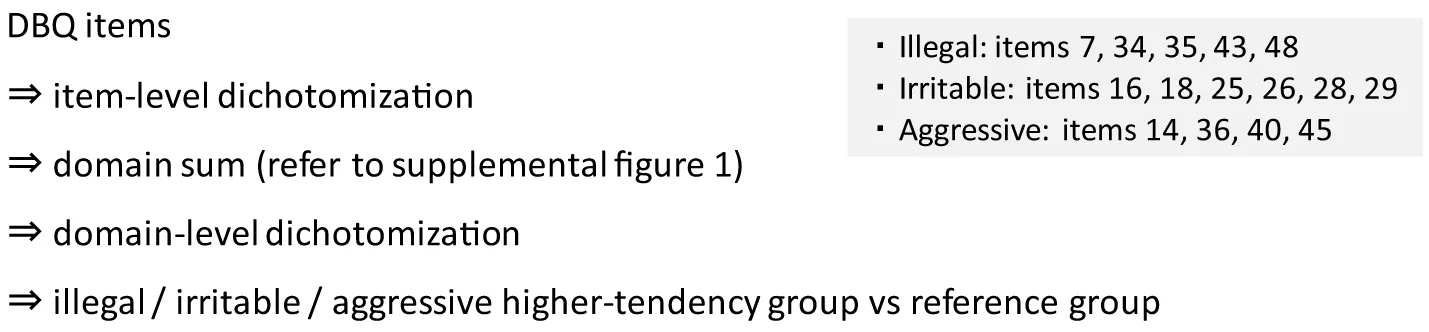
Schematic diagram of the derivation of three DBQ-based driving domains. Selected DBQ items were first dichotomized at the item level and then summed within each domain. The resulting domain scores were further dichotomized to define higher-tendency and reference groups for the illegal, irritable, and aggressive driving domains. This two-step dichotomization procedure was used instead of the original six-point DBQ responses to derive analyzable binary outcome variables for the main multivariable analyses.

As illustrated in Figure 2, selected DBQ items were first dichotomized at the item level and then summed within each domain. These domain scores were further dichotomized to define higher-tendency and reference groups. This approach was also intended to reduce the influence of highly skewed response distributions, as endorsement of hazardous driving behaviors was relatively infrequent in this community-based cohort. Item-specific thresholds were defined *a priori* to distinguish normative responses from those indicating that such behaviors occur at least occasionally. The thresholds of illegal, irritable, and aggressive tendencies were chosen to ensure the higher-tendency group comprised approximately 9.8, 13.0, and 9.9% of the respective sample: the threshold was set at two or more endorsed items for the illegal and aggressive domains, and three or more endorsed items for the irritable domain. Thus, these outcomes represent pragmatic indicators of relatively high hazardous driving tendencies within this cohort rather than clinically extreme phenotypes. This approach was chosen to facilitate robust classification of relatively infrequent hazardous driving behaviors while minimizing the influence of highly skewed response distributions.

Because most DBQ items showed highly skewed distributions with substantial floor effects, a two-step dichotomization procedure was adopted to obtain behaviorally meaningful domain classifications and to improve model stability. Continuous-score analyses were additionally performed as sensitivity analyses to evaluate the robustness of the findings.

### 2.4 MRI acquisition and volumetric processing

T1-weighted images were acquired using a 1.5-Tesla ECHELON Vega system (Hitachi, Tokyo, Japan) with 4-mm axial sections (TR = 9.2 ms; TE = 4.0 ms; TI = 1,000 ms; flip angle = 8°; FOV = 240 mm; matrix = 256 × 256). Because these data were obtained from routine clinical brain health examinations, MRI acquisition parameters reflected standard checkup protocols rather than research-optimized high-resolution volumetric imaging. All MR images underwent visual quality assessment to identify motion artifacts, major image degradation, and structural abnormalities that could compromise volumetric analysis. Regional brain volumes were estimated using SPM12 (https://www.fil.ion.ucl.ac.uk/spm/). Images were segmented into gray matter (GM), white matter (WM), and cerebrospinal fluid using a maximum a posteriori (MAP) approach. We utilized the DARTEL algorithm and a custom template derived from the IXI Dataset for high-dimensional nonlinear warping, which enhances sensitivity in detecting morphological variations. The Neuromorphometrics atlas in SPM12 was used for anatomical parcellation, with adjustments to reduce misclassification of periventricular white matter lesions as GM.

The volumes of 114 bilateral GM regions were calculated by summing the tissue densities within each parcellated region. The GM regional volumes were normalized by intracranial volume (ICV) to minimize inter-individual differences in overall brain size.

### 2.5 Statistical analysis

Separate multivariable logistic regression models were constructed for each driving domain. The binary classification (higher-tendency vs. reference) served as the dependent variable. Explanatory variables included age, sex, driving frequency, and z-transformed regional brain volumes. Given the exploratory nature of the study and the large number of regional variables, we performed bidirectional stepwise variable selection based on the Akaike Information Criterion (AIC) to derive parsimonious models.

Model robustness and variable stability were assessed using leave-one-out cross-validation (LOOCV). In each iteration, one participant was omitted, and the entire model-building process—including variable selection—was repeated. We recorded the selected variables, their associations, and classification performance. Stability and performance metrics are summarized in Tables 2, 3, respectively. Model discrimination was quantified using the area under the receiver operating characteristic curve (AUC), sensitivity, specificity, positive predictive value, and negative predictive value.

**Table 2:** Final multivariable logistic regression models for DBQ-derived hazardous driving tendencies, including variance inflation factors and direction of associations.

	Variables	OR	95%CI	*P*-values	VIF
Illegal					
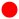	sex	3.33	(2.00, 5.56)	< 0.001	1.29
	R_accumb	1.19	(0.95, 1.49)	0.131	1.15
	L_caud_mid_front	1.24	(0.95, 1.62)	0.112	1.69
	L_cuneus	1.25	(0.96, 1.62)	0.096	1.51
	L_inf_temp	1.26	(0.99, 1.60)	0.063	1.36
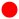	L_lat_occ	1.37	(1.06, 1.75)	0.015	1.5
	R_lat_occ	0.81	(0.63, 1.05)	0.116	1.49
	R_medial_orbfront	0.78	(0.57, 1.06)	0.118	2.07
	R_pars_orbit	0.83	(0.65, 1.07)	0.147	1.33
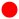	L_postcentral	1.33	(1.02, 1.74)	0.036	1.56
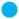	L_precentral	0.65	(0.47, 0.89)	0.007	2.16
	L_precuneus	0.77	(0.57, 1.03)	0.082	1.97
	L_sup_front	1.33	(0.98, 1.82)	0.067	2.22
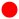	L_insula	1.66	(1.03, 2.70)	0.039	5.08
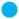	R_insula	0.54	(0.34, 0.87)	0.011	4.65
15.6-7.4,-1.3498pt	R_paracentral	1.2	(0.93, 1.54)	0.153	1.47
Irritable					
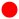	sex	3.59	(2.26, 5.70)	< 0.001	1.31
	age	0.98	(0.95, 1.00)	0.07	1.63
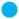	L_thal	0.75	(0.59, 0.95)	0.016	1.48
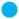	R_caud	0.76	(0.60, 0.95)	0.015	1.30
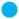	L_accumb	1.29	(1.05, 1.60)	0.017	1.29
	R_caud_mid_front	0.84	(0.67, 1.04)	0.113	1.36
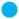	R_entorhinal	0.79	(0.63, 0.99)	0.037	1.35
	L_fusiform	1.27	(0.94, 1.73)	0.122	2.68
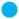	L_inf_temp	0.71	(0.53, 0.94)	0.016	2.21
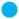	R_lat_occ	0.79	(0.63, 0.99)	0.041	1.43
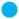	L_lingual	0.7	(0.53, 0.94)	0.018	2.36
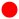	R_lingual	1.4	(1.05, 1.87)	0.022	2.26
	L_mid_temp	1.41	(0.98, 2.02)	0.064	3.58
	R_mid_temp	0.76	(0.53, 1.07)	0.119	3.36
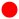	L_parahipp	1.33	(1.04, 1.70)	0.023	1.64
	R_postcentral	1.23	(0.98, 1.55)	0.07	1.53
	R_precuneus	1.23	(0.94, 1.60)	0.127	2.03
	R_sup_pariet	0.83	(0.65, 1.07)	0.152	1.64
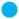	L_supramarg	0.71	(0.54, 0.94)	0.015	2.02
15.6-7.4,-1.3498pt	L_trans_temp	1.35	(1.08, 1.70)	0.009	1.50
Aggressive					
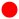	sex	9.44	(5.30, 16.82)	< 0.001	1.09
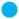	age	0.97	(0.95, 1.00)	0.037	1.33
	R_caud	0.73	(0.57, 0.95)	0.017	1.32
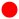	L_put	1.36	(1.02, 1.81)	0.036	1.74
	R_pal	0.78	(0.59, 1.02)	0.066	1.58
	L_accumb	1.25	(0.99, 1.58)	0.059	1.17
	L_cuneus	0.77	(0.59, 1.02)	0.065	1.61
	R_cuneus	1.28	(0.96, 1.70)	0.095	1.78
	L_inf_temp	0.75	(0.56, 1.01)	0.055	1.88
	R_inf_temp	1.34	(0.99, 1.81)	0.061	2.01
	L_lat_occ	1.26	(0.97, 1.63)	0.087	1.59
	L_lingual	0.74	(0.54, 1.01)	0.057	2.24
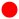	R_lingual	1.57	(1.14, 2.17)	0.006	2.29
	R_pericalcarine	0.78	(0.60, 1.00)	0.052	1.41
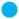	R_parahipp	0.77	(0.59, 1.00)	0.050	1.62

The table presents the final multivariable logistic regression models for the illegal, irritable, and aggressive driving domains. Candidate explanatory variables included age, sex, driving frequency, and z-transformed regional brain volume measures. Variables retained in the final models were selected using bidirectional stepwise selection based on the Akaike information criterion. For each retained variable, odds ratios, 95% confidence intervals, p values, and variance inflation factors (VIFs) are presented. Red and blue circles indicate positive and negative associations, respectively, between each explanatory variable and the DBQ-derived hazardous driving tendency. These results should be interpreted as findings from an exploratory model-building analysis. DBQ, Driving Behavior Questionnaire; OR, odds ratio; CI, confidence interval; VIF, variance inflation factor. Regional brain volume measures were standardized as z-scores before modeling. Red circles indicate positive associations (OR > 1), and blue circles indicate negative associations (OR < 1).

**Table 3:** Stability of variable selection and direction of association under leave-one-out cross-validation.

	Variables	Number of significance	Max OR	Min OR
Illegal				
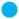	L_precentral	1,070	0.74	0.62
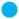	R_insula	1,070	0.72	0.48
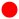	sex	1,070	4.61	3.13
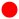	L_lat_occ	1,051	1.43	1.26
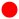	L_postcentral	762	1.38	1.30
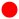	L_insula	712	1.83	1.62
	L_sup_front	274	1.52	1.34
	L_precuneus	30	0.74	0.68
	R_lat_occ	19	0.76	0.74
	L_inf_temp	18	1.30	1.27
	L_amyg	8	1.32	1.28
	R_accumb	4	1.31	1.28
	R_pars_orbit	2	0.77	0.74
	R_medial_orbfront	2	0.71	0.71
	L_caud_mid_front	2	1.35	1.32
15.6-7.2,-1.3498pt	R_paracentral	1	1.29	1.29
Irritable				
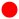	L_accumb	1,070	1.36	1.27
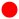	L_trans_temp	1,070	1.44	1.29
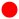	sex	1,070	4.24	3.37
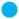	R_caud	1,070	0.78	0.72
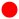	R_lingual	1,069	1.53	1.34
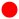	L_parahipp	1,067	1.43	1.28
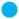	L_lingual	1,067	0.76	0.66
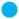	L_thal	1,067	0.80	0.67
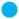	L_supramarg	1,066	0.76	0.68
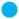	L_inf_temp	1,012	0.78	0.66
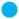	R_entorhinal	950	0.80	0.75
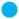	R_lat_occ	919	0.80	0.75
	age	109	0.98	0.97
	R_postcentral	35	1.28	1.24
	L_mid_temp	34	1.51	1.44
	R_inf_pariet	7	0.74	0.71
	R_parahipp	3	1.33	1.31
	L_insula	1	0.74	0.74
	L_caud_ant_cing	1	1.34	1.34
	R_caud_mid_front	1	0.79	0.79
15.6-7.4,-1.3498pt	R_rost_mid_front	1	1.41	1.41
Aggressive				
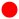	R_lingual	1,070	1.69	1.35
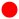	sex	1,070	11.19	8.21
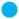	R_caud	1,070	0.75	0.65
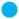	age	1,044	0.97	0.96
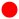	L_put	905	1.41	1.33
	R_parahipp	231	0.77	0.72
	L_lingual	186	0.74	0.68
	R_inf_temp	181	1.48	1.36
	R_pal	174	0.76	0.65
	L_inf_temp	165	0.75	0.72
	R_cuneus	122	1.49	1.32
	R_pericalcarine	68	0.77	0.75
	L_accumb	21	1.28	1.26
	L_cuneus	10	0.76	0.73
	L_hippo	4	1.37	1.33
	R_thal	1	1.89	1.89
	L_lat_occ	1	1.35	1.35

**Figure 3:**
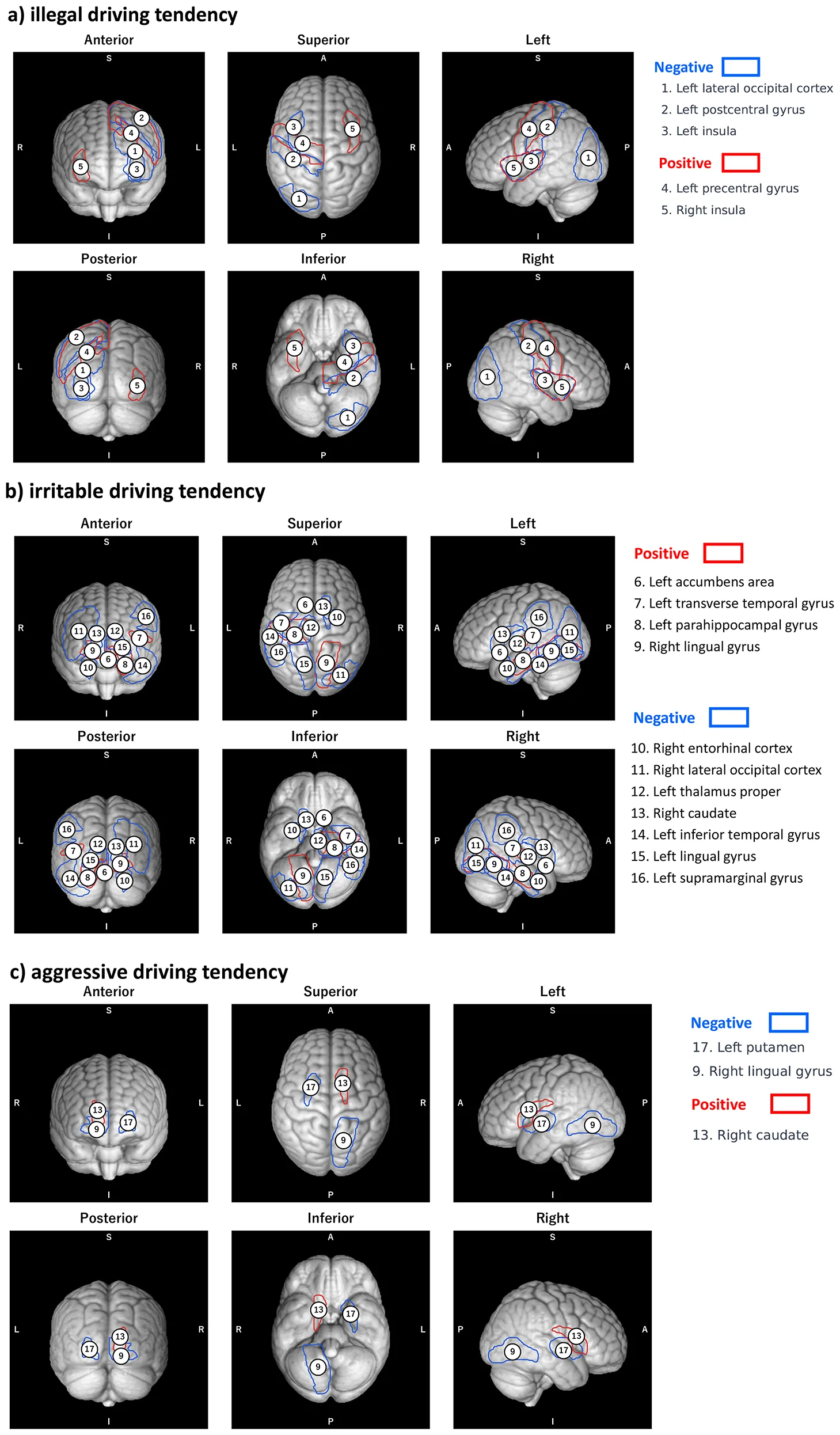
Three-dimensional visualization of representative brain regions associated with DBQ-derived hazardous driving tendencies. Regional brain areas retained in the final multivariable exploratory models are displayed on a standardized three-dimensional brain surface for **(a)** illegal, **(b)** irritable, and **(c)** aggressive driving domains. This figure is intended to illustrate the spatial distribution of the principal neuroanatomical correlates identified for each domain, rather than to imply voxel-wise localization or direct causal effects of individual regions. Red and blue indicate positive and negative associations, respectively, between regional brain volume and DBQ-derived hazardous driving tendency. Irritable and aggressive driving showed partial overlap, particularly in the right caudate and right lingual regions, whereas illegal driving showed a more distinct pattern involving insular, sensorimotor, and lateral occipital regions. The regions are presented as a visual summary of the final domain-specific models and should be interpreted in conjunction with the regression results and cross-validation stability reported in the text. S, superior; A, anterior; I, inferior; P, posterior; R, right; L, left.

The table summarizes leave-one-out cross-validation (LOOCV)-based stability of variable selection for the illegal, irritable, and aggressive driving domains. For each retained variable, the frequency of statistical significance across LOOCV iterations and the corresponding range of odds ratios are presented. Red and blue circles indicate positive and negative associations, respectively. Variables retained in more than 50% of LOOCV iterations are visually emphasized and correspond to the representative regions shown in Figure 3.

LOOCV, leave-one-out cross-validation; OR, odds ratio. Regional brain volume measures were standardized as z-scores before modeling. Red circles indicate positive associations (OR > 1), and blue circles indicate negative associations (OR < 1). Dashed lines indicate more than the half of 1,070 in the numbers of siginificant. Three dimentional imagings of the gray matter regions over the dashed line are presented in Figure 3.

## 3 Results

### 3.1 Distribution of DBQ-derived domain scores

The distributions of summed dichotomized scores for the illegal, irritable, and aggressive domains were notably right-skewed. Most participants were concentrated at lower scores, with relatively few endorsing multiple hazardous driving behaviors (Supplementary Figure 1).

### 3.2 Participant characteristics

Table 4 summarizes the demographic characteristics, driving frequency, intracranial volume, and global gray matter volume of the study participants. Across all three domains, the higher-tendency groups contained a substantially greater proportion of men than their respective reference groups. Mean age was slightly lower in the irritable and aggressive higher-tendency groups compared to the reference groups, whereas median driving frequency remained similar across all groups. In addition, modest differences in global brain volume measures were observed between the higher-tendency and reference groups. Participants with higher irritable and aggressive driving tendencies tended to show slightly larger TBV than their respective reference groups, whereas this tendency was not observed for illegal driving (Table 4). Because regional brain volumes were normalized by intracranial volume prior to statistical modeling, these global volumetric differences were not the primary focus of the present analyses.

**Table 4:** Participant characteristics according to DBQ-derived hazardous driving domains.

	>Illegal	Non-illegal	Irritable	Non-irritable	Aggressive	Non-aggressive
*N*	105	965	139	931	106	964
Male, *n* (%)	75 (71.4)	427 (44.2)	100 (71.9)	402 (43.2)	90 (84.9)	412 (42.7)
Age, years	53.9 ± 8.7	53.2 ± 9.1	52.1 ± 7.8	53.5 ± 9.2	52.1 ± 9.1	53.4 ± 9.0
DF, median	4	4	4	4	4	4
TBV, cm^3^	1,115.7 ± 7.6	1,133.9 ± 7.9	1,144.0 ± 6.5	1,135.2 ± 7.9	1,146 ± 6.6	1,134.7 ± 8.8
GMV, cm^3^	525.4 ± 4.8	522.7 ± 3.9	519.8 ± 4.1	523.7 ± 3.2	521.6 ± 3.7	524.2 ± 4.3

Participant characteristics are shown separately for the higher-tendency and reference groups in the illegal, irritable, and aggressive driving domains. Data are presented as mean ± standard deviation unless otherwise indicated. Driving frequency (DF) is shown as the median. TBV indicates total brain volume, and GMV indicates gray matter volume.

DBQ, Driving Behavior Questionnaire; DF, driving frequency; GMV, gray matter volume; TBV, total brain volume.

### 3.3 Final multivariable models

Odds ratios, 95% confidence intervals, and *p*-values for all retained variables of multiple logistic analyses are presented in Table 2. The final multivariable models are summarized as following.

- **Illegal driving:** the final model retained male sex, left lateral occipital volume, left postcentral volume, left precentral volume, and bilateral insular volumes. Male sex was associated with higher odds of illegal driving. Regarding brain morphology, smaller left precentral and right insular volumes were significantly associated with higher illegal driving tendencies while larger left lateral occipital, postcentral, and insular volumes were significantly associated with higher illegal driving tendencies.
- **Irritable driving:** the final model retained male sex, left thalamus, right caudate, left accumbens, right entorhinal cortex, left inferior temporal cortex, right lateral occipital cortex, bilateral lingual gyri, left parahippocampal gyrus, left supramarginal gyrus, and left transverse temporal gyrus. Male sex showed a positive association with irritable driving. Smaller right caudate, entorhinal cortex, and lateral occipital gyrus volumes and left thalamus, inferior temporal gyrus, lingual gyrus, and supramarginal gyrus volumes were significantly associated with higher illegal driving tendencies while larger right lingual and left accumbens, parahippocampal gyrus, and transverse temporal gyrus volumes were associated with higher irritable driving tendencies.
- **Aggressive driving:** the final model included male sex, younger age, right caudate, left putamen, right lingual gyrus, and right parahippocampal gyrus. Male sex and younger ages were positively associated with aggressive driving. Smaller right caudate and parahippocampal gyrus volume were associated with higher aggressive driving tendencies while larger left putamen and right lingual volume were associated with higher aggressive driving tendencies.

### 3.4 Cross-validation stability and model performance

LOOCV-based variable selection stability is summarized in Table 3. Male sex was selected in 100% of LOOCV iterations across all domains. The right caudate and right lingual regions were repeatedly selected in both irritable and aggressive models. In contrast, illegal driving was characterized by repeated selection of insular, sensorimotor, and lateral occipital regions. Overall LOOCV performance metrics are detailed in Table 5. Representative brain regions significantly identified in the final models are illustrated in Figure 3.

**Table 5:** Leave-one-out cross-validation performance of the final multivariable models for the three DBQ-derived driving domains.

Illegal	Mean ±S.D.	Irritable	Mean ±S.D.	Aggressive	Mean ±S.D.
Training accuracy	0.9023 ± 0.0025	Training accuracy	0.8732 ± 0.0021	Training accuracy	0.9005 ± 0.0036
Test accuracy	0.8572 ± 0.0018	Test accuracy	0.85761± 0.0015	Test accuracy	0.8981 ± 0.0074
Cross-validated AUC	0.7345 ± 0.0035	Cross-validated AUC	0.7568 ± 0.0003	Cross-validated AUC	0.7963 ± 0.0011
Sensitivity	0.6536 ± 0.0057	Sensitivity	0.7069 ± 0.0157	Sensitivity	0.7401 ± 0.0122
Specificity	0.7193 ± 0.0049	Specificity	0.6993 ± 0.0049	Specificity	0.7467 ± 0.0017
PPV	0.2043 ± 0.0014	PPV	0.2602 ± 0.0089	PPV	0.2437 ± 0.0093
NPV	0.9505 ± 0.0004	NPV	0.9431 ± 0.0023	NPV	0.9632 ± 0.0010

The table reports the performance of the final multivariable logistic regression models for the illegal, irritable, and aggressive driving domains under leave-one-out cross-validation (LOOCV). Reported values are presented as mean ± standard deviation and include training accuracy, test accuracy, cross-validated area under the receiver operating characteristic curve (AUC), sensitivity, specificity, positive predictive value (PPV), and negative predictive value (NPV). These metrics provide an internal assessment of discrimination and classification performance under LOOCV. AUC, area under the receiver operating characteristic curve; DBQ, Driving Behavior Questionnaire; LOOCV, leave-one-out cross-validation; NPV, negative predictive value; PPV, positive predictive value.

**Illegal driving:** Mean cross-validated AUC was 0.735 ± 0.004, with a sensitivity of 0.654 and specificity of 0.719.

**Irritable driving:** Mean AUC was 0.757 ± 0.000, with a sensitivity of 0.707 and specificity of 0.699.

**Aggressive driving:** Mean AUC was 0.796 ± 0.001, with a sensitivity of 0.740 and specificity of 0.747.

Across all models, performance was characterized by moderate discriminatory ability, high negative predictive values (NPVs > 0.94), and relatively low positive predictive values (PPVs). Although the models demonstrated moderate discrimination under LOOCV, positive predictive values remained relatively low, indicating limited performance for individual-level classification.

### 3.5 Sensitivity analyses using continuous DBQ scores

Sensitivity analyses using the original continuous DBQ domain scores were performed using multiple linear regression models (Supplementary Tables S2–S4). Overall, demographic associations were largely consistent with the primary analyses, whereas regional volumetric associations were substantially less stable across modeling approaches. Male sex remained significantly associated with all three hazardous driving domains, and younger age remained associated with aggressive driving. In contrast, regional volumetric associations were generally weaker and showed limited directional concordance with the primary dichotomized analyses. Detailed results are presented in Supplementary Tables S2–S4.

## 4 Discussion

In this study of 1,070 neurologically healthy adults, DBQ-derived illegal, irritable, and aggressive driving tendencies were associated with partially distinct yet overlapping regional brain volumetric patterns. Across all domains, male sex was consistently linked to higher hazardous driving tendencies, while younger age was specifically associated with aggressive driving. Internal validation using LOOCV supported the relative stability of several core associations, particularly involving the right caudate for irritable and aggressive driving and insular-sensorimotor regions for illegal driving. Together, these findings suggest that different forms of hazardous driving may reflect partially distinct neurobehavioral processes rather than a single underlying mechanism.

A primary finding is that the three DBQ domains exhibited divergent neuroanatomical profiles. Illegal driving was primarily associated with the insular, precentral, postcentral, and lateral occipital regions. In contrast, irritable driving showed a broader pattern involving thalamic, striatal, temporolimbic, and posterior cortical regions, while aggressive driving displayed a more compact pattern centered on the caudate, putamen, and right lingual region. The partial overlap between irritable and aggressive driving—specifically in the right caudate and right lingual gyrus—suggests that emotionally driven and hostile behaviors may share neurobehavioral substrates related to response selection, self-regulation, and visual-contextual processing (Dang et al., 2016; Elliott et al., 2023; Love and Nicolls, 2025).

These findings are consistent with the view that hazardous driving is not a unitary phenomenon but may comprise multiple behaviorally distinct tendencies. The partially divergent neuroanatomical profiles observed across the three DBQ-derived domains support the utility of distinguishing between deliberate rule violation, frustration-related driving, and hostile or competitive driving when examining the neurobehavioral correlates of hazardous driving (Deffenbacher et al., 2001). Even within self-reported maladaptive behaviors, different tendencies map onto distinct neural constellations. From a traffic safety perspective, this distinction is critical; interventions may need to be tailored depending on whether unsafe driving is driven by deliberate rule violation, frustration/impatience, or interpersonal hostility (Reason et al., 1990; de Winter and Dodou, 2010; Cordazzo et al., 2014; Parker et al., 1995; Love and Nicolls, 2025).

These findings should not be interpreted as indicating that the volume of any single brain region directly determines hazardous driving behavior. Rather, regional volumetric variation likely reflects differences in distributed neural systems involved in salience detection, cognitive control, emotional regulation, and behavioral selection (Menon and Uddin, 2010; Dang et al., 2016; Elliott et al., 2023). Accordingly, the identified regions are best viewed as markers of network-level variation rather than direct causal determinants of unsafe driving.

The predictive performance of our models warrants cautious interpretation. While AUC values were acceptable and internally stable, the low positive predictive values likely reflect the low prevalence of high-tendency cases in this community-based cohort rather than poor discriminatory power. Consequently, these should be viewed as exploratory association models rather than clinically ready screening tools (de Winter and Dodou, 2010).

Importantly, these findings may have implications beyond the neuroanatomical characterization of driving style. Previous studies have reported associations between DBQ-derived hazardous driving behaviors and crash involvement (Reason et al., 1990; de Winter and Dodou, 2010; Cordazzo et al., 2014; Parker et al., 1995). Within this context, the present findings suggest that individual differences in brain structure may contribute to behavioral tendencies that have previously been linked to traffic safety outcomes. However, because crash outcomes were not examined in the present study, any relationship between the identified neuroanatomical patterns and actual crash risk remains speculative and requires direct investigation in future longitudinal studies.

To better understand the neurobehavioral basis of these findings, the identified regional patterns can be interpreted in terms of three partially distinct functional systems related to risk regulation, emotional reactivity, and habitual behavioral responding.

Illegal driving tendencies were primarily associated with sensorimotor and insular regions, including the precentral gyrus, postcentral gyrus, and insula. These regions are involved in motor execution, sensory feedback, and salience-based risk monitoring (Menon and Uddin, 2010). The insula has previously been implicated in salience processing, interoceptive awareness, and behavioral regulation in functional neuroimaging studies (Menon and Uddin, 2010). Therefore, the present structural MRI findings are consistent with the possibility that illegal driving tendencies may involve neural systems related to risk monitoring and behavioral restraint. However, because the present study examined regional brain volumes rather than neural activity, these functional interpretations remain speculative and should be regarded as hypotheses informed by prior literature rather than direct conclusions from the current data. Individuals exhibiting such tendencies may recognize potentially hazardous traffic situations but show a reduced propensity to translate perceived risk into adaptive behavioral control.

In contrast, irritable driving tendencies were associated with the caudate and limbic-related structures, including the nucleus accumbens, parahippocampal region, and thalamus. The caudate has been linked to impulsivity and behavioral regulation (Dang et al., 2016), whereas the nucleus accumbens plays a central role in reward processing and motivational behavior (Knutson et al., 2001). The parahippocampal region contributes to contextual evaluation and memory-guided behavioral responses (Aminoff et al., 2013), while the thalamus serves as an important hub integrating emotional, cognitive, and attentional information across distributed neural networks (Sherman, 2016). Collectively, these findings suggest that irritable driving may be characterized by heightened emotional responsiveness to traffic-related stressors and a tendency for frustration-related emotional states to influence behavioral responses during driving situations. Individuals exhibiting such tendencies may be more likely to react to traffic congestion, unexpected events, or the behavior of other road users with emotionally mediated responses, including impatience and irritation.

Aggressive driving exhibited a more focused frontostriatal pattern involving the caudate and putamen. Both structures are key components of basal ganglia circuits involved in action selection, reinforcement learning, and the development of habitual behavioral responses. The caudate has been implicated in behavioral regulation and goal-directed action selection (Dang et al., 2016; Balleine and O'Doherty, 2010), whereas the putamen plays a central role in habit formation and automatic action execution (Balleine and O'Doherty, 2010; Graybiel, 2008). This neuroanatomical profile raises the possibility that aggressive driving may reflect a tendency for emotionally triggered reactions to become translated into relatively automatic and repeatedly expressed behavioral patterns. In contrast to irritable driving, which may primarily reflect transient emotional responses to frustrating traffic situations, aggressive driving may involve more ingrained behavioral tendencies characterized by hostile, retaliatory, or confrontational actions toward other road users. Such behaviors may emerge when emotional arousal is coupled with habitual action-selection processes, resulting in the repeated expression of aggressive responses under perceived provocation.

From a broader traffic-safety perspective, these findings suggest that different hazardous driving styles may originate from distinct neurobehavioral processes related to risk regulation (illegal), emotional reactivity (irritable), and habitual aggressive response tendencies (aggressive). This interpretation is consistent with the view that illegal, irritable, and aggressive driving represent partially independent dimensions of hazardous driving behavior rather than manifestations of a single underlying trait. Understanding these differences may contribute to the development of more individualized approaches to driver assessment, behavioral intervention, and crash-risk prevention, while also providing an initial neuroanatomical framework for studying hazardous driving phenotypes.

Sensitivity analyses using the original continuous DBQ scores (Supplementary Table S1) generally identified similar neuroanatomical regions; however, the models were less stable and showed greater variability in variable selection. These findings support the use of the dichotomized domains in the present exploratory neuroimaging study while acknowledging the inherent loss of quantitative information associated with dichotomization.

An additional finding of the present study was that analyses based on the original continuous domain scores yielded less stable neuroanatomical models than those based on the dichotomized domains. Rather than indicating an absence of meaningful brain–behavior relationships, this instability is likely attributable to the highly skewed distributions and substantial floor effects of the original DBQ responses in this community-based cohort. Under such conditions, relatively small variations in low-frequency behavioral responses may disproportionately influence variable selection, thereby reducing model stability. In contrast, the dichotomized domains provided more stable identification of regional volumetric patterns while retaining the theory-driven classification framework used to distinguish the three hazardous driving phenotypes. Nevertheless, future studies should examine whether larger cohorts and alternative statistical or machine-learning approaches can more robustly model hazardous driving tendencies as continuous behavioral traits.

The modest differences observed in total brain volume deserve consideration. Although participants with higher hazardous driving tendencies tended to exhibit slightly larger TBV in some domains, regional volumetric analyses were performed after normalization for intracranial volume to minimize the influence of overall brain size. Consequently, the present findings primarily reflect relative regional volumetric variation rather than global brain size differences. Nevertheless, whether global brain volume itself contributes to hazardous driving tendencies warrants further investigation.

### 4.1 Limitations

Several limitations should be acknowledged. First, the study population consisted of Japanese adults attending routine health checkups and may therefore exhibit less behavioral variability than the general driving population, potentially underrepresenting individuals with more extreme hazardous driving tendencies. Second, the primary analyses relied on a two-step dichotomization procedure, which inevitably reduced information contained in the original six-point DBQ scales. Consistent with this concern, supplementary analyses using the original continuous domain scores yielded only partial concordance with the primary regional findings, suggesting that some neuroanatomical associations may be sensitive to the operationalization of hazardous driving outcomes.

In addition, the three hazardous driving domains examined in this study were theoretically derived from selected DBQ items rather than established through formal psychometric validation procedures. Although the domains were specified a priori and were designed to capture conceptually distinct forms of hazardous driving behavior, alternative item groupings may also be plausible. Future studies should evaluate the reproducibility, construct validity, and psychometric properties of these theory-driven domains in independent populations.

Third, although bidirectional stepwise AIC-based model selection provided a practical approach for exploratory analysis of a relatively large number of regional brain volumetric measures, such procedures remain susceptible to overfitting, model instability, and optimistic effect estimates. Model robustness was therefore assessed using leave-one-out cross-validation, which demonstrated generally consistent directions of the identified brain–behavior associations across validation iterations. Nevertheless, the selected brain regions should be regarded as candidate neuroanatomical correlates requiring confirmation in independent cohorts using more advanced statistical and machine-learning approaches.

Fourth, the use of 1.5-T MRI with 4-mm slice thickness may have reduced sensitivity for detecting subtle volumetric differences compared with contemporary high-resolution volumetric imaging protocols (Putra et al., 2023; Ashburner and Friston, 2000). Although internal validation supported the stability of the identified associations, external validation in independent cohorts will be necessary to establish their robustness and generalizability.

Fifth, although LOOCV provides an internal assessment of model stability, it does not replace independent external validation or repeated resampling approaches such as bootstrap validation. Because the present analyses were based on a historical dataset for which the original analysis dataset is no longer available, additional resampling analyses could not be performed retrospectively. Therefore, the present findings should be regarded as exploratory and hypothesis-generating.

Another limitation is that several potentially important confounding variables were unavailable in this retrospective clinical cohort. Because the primary purpose of the brain health checkup program was the detection of structural cerebrovascular abnormalities rather than comprehensive neuropsychological or behavioral phenotyping, standardized cognitive assessments, educational attainment, psychiatric symptoms, alcohol consumption, medication use, socioeconomic status, and lifetime driving exposure were not routinely collected. Consequently, residual confounding cannot be excluded, and the observed brain–behavior associations should be interpreted as associations rather than evidence of direct neuroanatomical mechanisms. Future prospective studies incorporating comprehensive cognitive, behavioral, and lifestyle assessments will be important to clarify the independent contribution of brain structure to hazardous driving behavior.

Although the present models demonstrated moderate discriminative performance, they should not be interpreted as clinically applicable tools for individual-level prediction or screening. Rather, the primary contribution of this study is the identification of initial neuroanatomical framework and the generation of biologically informed hypotheses regarding distinct hazardous driving phenotypes. Translation to individualized prediction will require larger cohorts, high-resolution MRI, external validation, and more advanced analytical approaches.

Sex was consistently identified as one of the strongest predictors across all hazardous driving domains. Although sex was included as a covariate in all multivariable models, sex-stratified analyses were beyond the scope of the present study. Future studies in larger and more diverse cohorts should investigate sex-specific neuroanatomical patterns and potential interactions between sex and hazardous driving phenotypes.

Finally, because the present study was based on cross-sectional structural MRI, the observed regional volumetric associations should be regarded as anatomically informed hypotheses rather than direct evidence of neural activity or psychological mechanisms. Accordingly, interpretations regarding risk regulation, emotional reactivity, behavioral control, and habitual responding remain speculative and are based primarily on previous neuroimaging literature. Future multimodal neuroimaging studies incorporating functional MRI and longitudinal designs will be necessary to validate these proposed neurobehavioral mechanisms.

Despite these limitations, the present study benefits from a relatively large community-based sample, domain-specific decomposition of hazardous driving tendencies, and systematic internal validation. Accordingly, the findings should be regarded as hypothesis-generating rather than definitive evidence of specific neurobiological mechanisms or clinically applicable biomarkers of unsafe driving. Instead, this study provides an initial neuroanatomical framework suggesting that illegal, irritable, and aggressive driving tendencies may be associated with partially distinct neural systems related to risk regulation, emotional reactivity, and habitual aggressive responding. Although confirmation in independent cohorts using contemporary statistical and machine-learning approaches will be essential, these findings provide a foundation for future research linking brain structure, hazardous driving behavior, and traffic crash risk, and may ultimately support more individualized approaches to driver assessment and traffic-safety interventions.

## 5 Conclusion

In this large community-based cohort of neurologically healthy adults, distinct DBQ-derived hazardous driving tendencies were associated with partially divergent yet overlapping regional brain volumetric patterns. Illegal, irritable, and aggressive driving exhibited different neuroanatomical profiles, supporting the concept that hazardous driving is not a unitary behavioral construct but may involve partially distinct neurobehavioral systems related to risk regulation, emotional reactivity, and habitual aggressive responding. Although these findings should be regarded as hypothesis-generating and require confirmation in independent cohorts using high-resolution MRI and contemporary analytical approaches, they provide an initial neuroanatomical framework for future research linking brain structure, hazardous driving behavior, and traffic crash risk.

## Data Availability

The raw data supporting the conclusions of this article will be made available by the authors, without undue reservation.
