# Supplementary material for: Distinct neuroanatomical correlates of DBQ-derived illegal, irritable, and aggressive driving tendencies in healthy adults: an exploratory structural MRI study
Source: Front Hum Neurosci. 2026 Aug 28;20:1907749. doi: 10.3389/fnhum.2026.1907749 (PMC13561989; doi:10.3389/fnhum.2026.1907749)
Supplement: Supplementary file 1 [file Data_Sheet_1.docx]

**Supplementary Material**

**Supplementary Figure S1. Distribution of summed dichotomized scores for the three DBQ-derived driving domains.**

Summed dichotomized scores are shown for the illegal, irritable, and aggressive driving domains. For each domain, selected DBQ items were first dichotomized and then summed within domain. The distributions were right-skewed, with most participants concentrated at lower scores and relatively few participants showing multiple endorsed hazardous driving behaviors. These distributional patterns provide context for the domain-level dichotomization used to define higher-tendency and reference groups in the main analyses.

**Supplementary Table S1. DBQ Items Selected for Theory-Driven Hazardous Driving Domains**

| Domain | DBQ Item No. | Original DBQ Item | Behavioral rationale |
| --- | --- | --- | --- |
| **Illegal** | 7 | Deliberately disregarding the speed limit late at night or early in the morning. | Intentional violation of traffic regulations despite awareness of legal restrictions and safety implications. |
|  | 34 | I ran a red light while driving on an empty road late at night. | Deliberate disregard of a traffic control device when enforcement or immediate danger is perceived to be low. |
|  | 35 | I deliberately drove the wrong way on a deserted one-way street. | Conscious violation of traffic laws for personal convenience. |
|  | 43 | I knew my blood alcohol level was above the legal limit, but I drove home from a party or restaurant. | Intentional violation of legal requirements despite awareness of increased crash risk. |
|  | 48 | I ignored a “Stop” sign and narrowly avoided a collision with a vehicle that had the right of way. | Deliberate failure to comply with mandatory traffic regulations. |
| **Irritable** | 16 | Failing to yield to a bus that was signaling to move. | Impatience-driven behavior reflecting frustration with perceived delays imposed by other road users. |
|  | 18 | Overtaking a slow-moving vehicle on a highway by using the inner lane or shoulder. | Risk-taking behavior motivated by impatience and intolerance of reduced traffic flow. |
|  | 25 | Becoming frustrated with a slow driver in the passing lane and overtaking from the inside. | Frustration-induced response to perceived obstruction by another driver. |
|  | 26 | Approaching the vehicle in front extremely closely or flashing headlights to signal it to go faster or move out. | Emotionally driven expression of impatience toward slower traffic. |
|  | 28 | I was driving slowly behind a car on a single-lane road and became impatient, feeling the urge to overtake despite the dangerous situation. | Internal experience of frustration and urgency leading to hazardous decision tendencies. |
|  | 29 | I took a gamble and crossed the street as the light was turning red. | Risk-taking behavior arising from impatience and unwillingness to tolerate delay. |
| **Aggressive** | 14 | Competing with oncoming traffic to get through a narrow section of a road or road with obstacles. | Competitive behavior involving interpersonal contest for road priority. |
|  | 36 | I got angry at another driver’s actions and chased after them to complain. | Direct retaliatory behavior toward another road user motivated by hostility. |
|  | 40 | I engaged in a near-car race with another driver. | Competitive and dominance-oriented interaction with another driver. |
|  | 45 | I have a strong dislike for certain types of drivers and tend to show hostility towards them at every opportunity. | Persistent hostile attitude directed toward other road users. |

**Note.** Domains were defined *a priori* to represent theoretically distinct forms of hazardous driving behavior. The classification was based on the predominant behavioral characteristics expressed by each DBQ item rather than on exploratory factor analysis. Illegal driving reflects deliberate rule violations, irritable driving reflects frustration- and impatience-related hazardous behaviors, and aggressive driving reflects hostile, retaliatory, or competitive interactions with other road users. These domains were conceptualized as theoretically derived behavioral phenotypes for neuroanatomical investigation and were not intended to redefine the psychometric factor structure of the original DBQ.

**Supplementary Table S2. Sensitivity analyses using the original continuous Illegal Driving scores.**

Standardized multiple-linear-regression coefficients for the continuous Illegal Driving scores (N =1070). Continuous-score models explained only a modest proportion of variance (adjusted R² ≈ 0.03–0.06). β are standardized for continuous predictors; sex is coded male = 1. Regional volumes were normalized by total brain volume and z-scored. SE, standard error; CI, confidence interval. *p < 0.05.

Significant covariates: Age (β=-0.035, lower); Sex (male=1) (β=0.425, higher); Driving frequency (β=0.074,higher). Significant regional associations: None.

**Supplementary Table S3. Sensitivity analyses using the original continuous Irritable Driving scores.**

Standardized multiple-linear-regression coefficients for the continuous Irritable Driving scores (N =1070). Continuous-score models explained only a modest proportion of variance (adjusted R² ≈ 0.03–0.06). β are standardized for continuous predictors; sex is coded male = 1. Regional volumes were normalized by total brain volume and z-scored. SE, standard error; CI, confidence interval. *p < 0.05.

Significant covariates: Age (β=-0.050, lower); Sex (male=1) (β=0.608, higher); Driving frequency (β=0.033,higher). Significant regional associations: L_lingual (β = −0.029, p = 0.032); R_entorhinal (β = 0.028, p = 0.014).

**Supplementary Table S4 Sensitivity analyses using the original continuous Aggressive Driving scores.**

Standardized multiple-linear-regression coefficients for the continuous Aggressive Driving scores (N =1070). Continuous-score models explained only a modest proportion of variance (adjusted R² ≈ 0.03–0.06). β are standardized for continuous predictors; sex is coded male = 1. Regional volumes were normalized by total brain volume and z-scored. SE, standard error; CI, confidence interval. *p < 0.05.

Significant covariates: Age (β=-0.052, lower); Sex (male=1) (β=0.559, higher); Driving frequency (β=0.030,higher). Significant regional associations: None.
